# Polatuzumab vedotin and CD79B: A study of efficacy in R‐CHOP‐resistant diffuse large B‐cell lymphoma

**DOI:** 10.1111/bjh.70185

**Published:** 2025-10-07

**Authors:** Nicolas Munz, Alberto J. Arribas, Roberta Bordone Pittau, Federico Simonetta, Georg Stussi, Francesco Bertoni

**Affiliations:** ^1^ Faculty of Biomedical Sciences Institute of Oncology Research, USI Bellinzona Switzerland; ^2^ SIB Swiss Institute of Bioinformatics Lausanne Switzerland; ^3^ Oncology Institute of Southern Switzerland Ente Ospedaliero Cantonale (EOC) Bellinzona Switzerland; ^4^ Division of Hematology, Department of Oncology Geneva University Hospitals, University of Geneva Geneva Switzerland; ^5^ Faculty of Medicine, Translational Research Centre in Onco‐Haematology University of Geneva Geneva Switzerland; ^6^ Faculty of Biomedical Sciences USI Lugano Switzerland

**Keywords:** antibody–drug conjugate, CD79B, disulfiram, DLBCL, loncastuximab tesirine, lymphoma, polatuzumab vedotin, R‐CHOP

To the Editor,

The standard treatment for patients affected by diffuse large B‐cell lymphoma (DLBCL) is still largely represented by the R‐CHOP regimen (rituximab, cyclophosphamide, doxorubicin, vincristine and prednisone).^1^ Only up to two‐thirds of treated patients are cured, indicating a too high fraction of patients still do not respond or relapse after a first R‐CHOP treatment.^1^ Patients with initial R‐CHOP treatment failure have poor prognoses and outcomes.^1^ Recent improvements in understanding DLBCL have led to a better refinement of disease classification and the development of new therapeutic strategies, including chimeric antigen receptor (CAR) T‐cell therapy, bispecific antibodies and antibody–drug conjugates (ADCs). Two ADCs are now approved for relapsed/refractory (R/R) DLBCL patients across different countries: loncastuximab tesirine and polatuzumab vedotin.^1^, ^2^ Loncastuximab tesirine is an anti‐CD19 antibody conjugated to the deoxyribonucleic acid (DNA)‐cross‐linking pyrrolobenzodiazepine (PBD) dimer warhead SG3199.^2^ Polatuzumab vedotin is an anti‐CD79B antibody conjugated to the microtubule‐disrupting monomethyl auristatin E (MMAE) as a payload.^1^, ^3^ The optimal sequencing of therapies for the improved management of DLBCL patients is an active field of research. We recently identified seven DLBCL models with reduced sensitivity to R‐CHOP (50 percent growth inhibitory concentration (IC_50_) values higher than the 75th percentile, i.e. 0.077 μg/mL).^4^ Four cell lines (Pfeiffer, U2932, SU‐DHL‐16, SU‐DHL‐2) also showed reduced sensitivity to loncastuximab tesirine.^4^ Thus, we exposed all the R‐CHOP‐resistant, alongside four R‐CHOP‐sensitive cell lines, to polatuzumab vedotin. In addition, we also treated REC1, a mantle cell lymphoma (MCL) cell line resistant to loncastuximab tesirine, its DNA‐damaging SG3199 warhead and other ADCs.^4^, ^5^, ^6^ All but two cell lines showed a dose–response sensitivity to polatuzumab vedotin, both derived from activated B‐cell‐like (ABC) DLBCL (Figure 1). One was the SU‐DHL‐2, resistant to R‐CHOP and loncastuximab tesirine.^4^ The other was the RCK8 with intermediate sensitivity to R‐CHOP and loncastuximab tesirine‐sensitive.^4^ The remaining six R‐CHOP‐resistant cell lines, including three with low sensitivity to loncastuximab tesirine and the MCL cell line resistant to multiple ADCs, responded to polatuzumab vedotin. Figure S1 shows the Spearman correlation analysis between the anti‐tumour activity of polatuzumab vedotin and the expression levels, measured via total ribonucleic acid (RNA)‐Seq, of its target CD79B. In this small and selected cell line panel, we observed no significant correlation, although the two cell lines with low sensitivity to the ADC were also the two models with the lowest *CD79B* expression. Previous studies have demonstrated that the SU‐DHL‐2 and RCK8 cell lines exhibit low levels of CD79B expression and are resistant to polatuzumab vedotin‐based treatment. In contrast, the SU‐DHL‐4 and U2932 cell lines display higher sensitivity to polatuzumab vedotin.^7^ Our results corroborate these findings by extending the analysis to an additional panel of DLBCL cell lines, thereby reinforcing the association between *CD79B* expression levels and therapeutic response.

**FIGURE 1:**
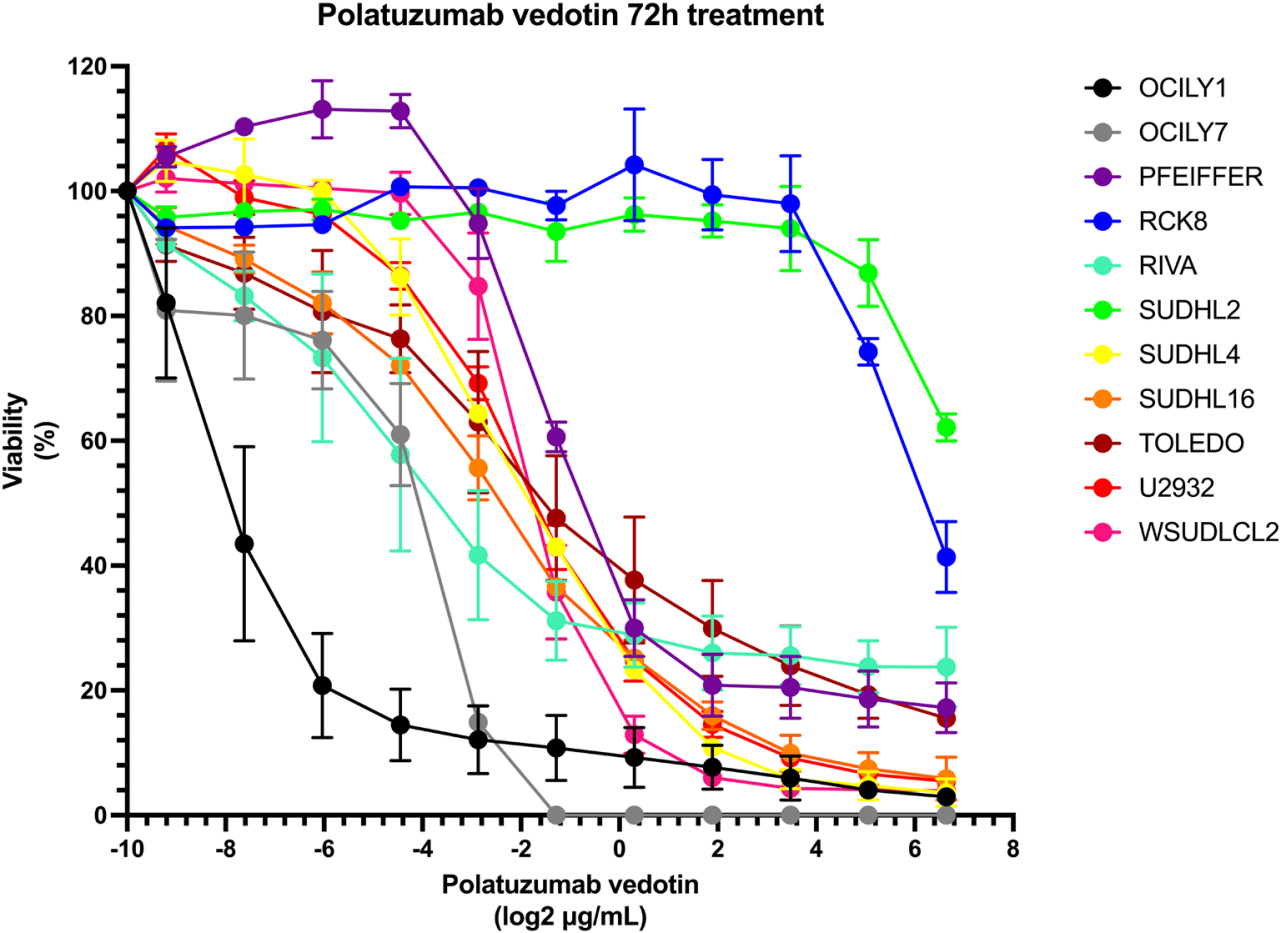
*In vitro* activity of polatuzumab vedotin in panel of lymphoma cell lines. Dose–response curve after 72 h of treatment. The observed sensitivity and resistance to polatuzumab vedotin were in line with two previous studies.^3^, ^7^ *Y*‐axis, percentage of proliferating cells compared to dimethyl sulfoxide (DMSO)‐treated cells. *X*‐axis, polatuzumab concentration.

Comparing RNA seq data of polatuzumab vedotin resistant (RCK‐8 and SU‐DHL‐2) and sensitive cell lines (all other DLBCL cell lines tested) using the limma pipeline revealed Aldehyde Dehydrogenase 1 Family Member L1 (*ALDH1L1*) as the second most upregulated gene in resistant cells (log_2_‐fold change = 11.06; adjusted *p* < 0.001) (Table S1). In the tested cell lines, we observed a significant negative correlation between *CD79B* and *ALDH1L1* expression (*p* = 0.0132; cor = −0.72, Figure S2). Notably, a negative correlation between *CD79B* and *ALDH1L1* was also observed in a cohort of DLBCL patients with publicly available expression data^8^ (*p* = 0.0221; cor = −0.13, Figure S3). These findings indicate that reduced *CD79B* expression is associated with increased *ALDH1L1* expression. Previous studies have demonstrated that the deregulation of ALDH1L1 can disrupt various cellular metabolic pathways.^9^, ^10^, ^11^ This change might lead to activation of alternative energy metabolism such as oxidative phosphorylation, producing adenosine triphosphate (ATP) from the reduced nicotinamide adenine dinucleotide (NADH) mediated by ALDH1L1.^10^ This metabolic shift might contribute to a reduced dependency on B cell receptor (BCR) signalling and CD79B expression, leading to reduced sensitivity to polatuzumab vedotin. Combining ALDH1L1 inhibitors with polatuzumab vedotin could provide synergistic anti‐lymphoma activity by concurrently impairing distinct metabolic vulnerabilities. Since ALDH1L1 is targeted by disulfiram,^10^ we combined disulfiram and polatuzumab vedotin in four DLBCL cell lines, selected for their different degrees of sensitivity to the ADC: two polatuzumab vedotin‐resistant (the ABC DLBCL SU‐DHL‐2 and RCK8) and two polatuzumab vedotin‐sensitive models (the ABC DLBCL RIVA and the GCB DLBCL SU‐DHL‐16). The combination determined an additive cytotoxic effect of 200–400 nM disulfiram to polatuzumab vedotin in all cell lines tested, not limited to polatuzumab vedotin‐resistant DLBCL cell lines with increased ALDH1L1 expression (Figure S4). Disulfiram exposure did not increase the surface expression of CD79B, CD19 or CD20 in SU‐DHL‐2 or SU‐DHL‐16 cells (Table S2).

The observed benefit of combining the ADC with disulfiram across all tested cell lines may be explained by disulfiram's multifaceted mechanisms of action. Disulfiram has been shown to induce intracellular reactive oxygen species, promote cell death and inhibit both proteasome activity and nuclear factor kappa‐light‐chain‐enhancer of activated B cells (NF‐κB) signalling,^12^ potentially modulating cellular sensitivity to the ADC.^13^ In addition, disulfiram can inhibit the p97 segregase, leading to proteotoxic stress.^14^ Under such conditions, activation of the integrated stress response supports microtubule‐mediated transport of misfolded proteins to the perinuclear space, where they are assembled into aggresomes and subsequently degraded through the ubiquitin–proteasome system during stress recovery.^15^ Thus, the combination of disulfiram‐induced proteotoxic stress with polatuzumab‐mediated microtubule disruption may synergistically impair the clearance of toxic protein aggregates, thereby amplifying apoptotic cell death.

These findings highlight disulfiram as a promising combination partner to enhance the activity of polatuzumab in multidrug‐resistant DLBCL and warrant further investigation. Moreover, ALDH1L1 expression may represent a predictive biomarker for response to polatuzumab, potentially in combination with other markers such as ABCG2, a well‐characterized ATP‐binding cassette transporter that was strongly upregulated in polatuzumab‐resistant DLBCL cell lines. These associations require confirmation in future studies.

In conclusion, our in vitro data show that the CD79B‐targeting polatuzumab vedotin is an active agent in DLBCL models with resistance to R‐CHOP and other ADCs. Lymphomas bearing low CD79B expression might benefit more from agents targeting CD19 or acting via different mechanisms of action. Our data also highlight the importance of developing novel therapies and drug combinations that can overcome the ability of certain lymphoma cells to resist chemotherapy and multiple ADCs.

## AUTHOR CONTRIBUTIONS

NM performed experiments and data mining, interpreted data and co‐wrote the manuscript. AJA interpreted data and provided advice. RBP and GS performed flow cytometry analyses. FS provided advice. FB designed the study, interpreted data, supervised the study and co‐wrote the manuscript. All authors reviewed and accepted the final version of the manuscript.

## CONFLICT OF INTEREST STATEMENT

Alberto J. Arribas: travel grant from Astra Zeneca and Floratek Pharma, consultant for PentixaPharm. Federico Simonetta: institutional consulting fees from BMS/Celgene, Incyte, Kite/Gilead; speaker fees from Kite/Gilead, Incyte; travel support from Kite/Gilead, Novartis, AstraZeneca, Neovii, Janssen; research funding from Kite/Gilead, Novartis, BMS/Celgene. Georg Stussi: travel grants from Novartis, Celgene, Roche; consultancy fee from Novartis; scientific advisory board fees from Bayer, Celgene, Janssen, Novartis; speaker fees from Gilead. Francesco Bertoni: institutional research funds from ADC Therapeutics, Bayer AG, BeiGene, Floratek Pharma, Helsinn, HTG Molecular Diagnostics, Ideogen AG, Idorsia Pharmaceuticals Ltd., Immagene, ImmunoGen, Menarini Ricerche, Nordic Nanovector ASA, Oncternal Therapeutics, Spexis AG; consultancy fee from BIMINI Biotech, Floratek Pharma, Helsinn, Immagene, Menarini, Vrise Therapeutics; advisory board fees to the institution from Novartis; expert statements provided to HTG Molecular Diagnostics; travel grants from Amgen, Astra Zeneca, iOnctura. The other authors have no conflicts of interest.

## Supporting information


Data S1.
