# Supplementary material for: Polatuzumab vedotin and CD79B: A study of efficacy in R‐CHOP‐resistant diffuse large B‐cell lymphoma
Source: Br J Haematol. 2025 Oct 7;207(6):2550–2. doi: 10.1111/bjh.70185 (PMC12710110; doi:10.1111/bjh.70185)
Supplement: Supplementary file 1 — Data S1. [file BJH-207-2550-s001.zip › bjh70185-sup-0001-supplementary revised copy.pdf]

Polatuzumab Vedotin and CD79B: A Study of Efficacy in R-CHOP-Resistant Diffuse Large B-Cell Lymphoma

Nicolas Munz [1], Alberto J. Arribas [1,2], Roberta Bordone Pittau [3], Federico Simonetta [4,5], Georg Stussi [3,6], Francesco Bertoni [1,3]

[1] Institute of Oncology Research, Faculty of Biomedical Sciences, USI, Bellinzona, Switzerland;
[2] SIB Swiss Institute of Bioinformatics, Lausanne, Switzerland;
[3] Oncology Institute of Southern Switzerland, Ente Ospedaliero Cantonale (EOC), Bellinzona, Switzerland;
[4] Division of Hematology, Department of Oncology, Geneva University Hospitals, University of Geneva, Geneva, Switzerland
[5] Translational Research Centre in Onco-Haematology, Faculty of Medicine, University of Geneva, Geneva, Switzerland
[6] Faculty of Biomedical Sciences, USI, Lugano, Switzerland.

Supplementary Materials

Supplementary Materials and Methods

Cell lines

Lymphoma cell lines were cultured according to the recommended conditions, as previously described [1]. All media were supplemented with fetal bovine serum (10% or 20%) and penicillin-streptomycin-neomycin (≈5,000 units penicillin, 5 mg streptomycin, and 10 mg neomycin/mL; Sigma). Human cell line identities were confirmed by short tandem repeat DNA fingerprinting using the Promega GenePrint 10 System kit (B9510). Cells were periodically tested for mycoplasma negativity using the MycoAlert Mycoplasma Detection Kit (Lonza).

Compounds

Polatuzumab vedotin was kindly provided by Roche, Switzerland. Disulfiram was purchased from MedChemExpress (Monmouth Junction, NJ, USA).

Cytotoxic activity in single and combination

Cells were seeded at the concentration of 20,000/per well in 96-well plates and exposed to polatuzumab vedotin (kindly provided by Roche, Switzerland) in a 1/3 dilution series ranging from 1.7 ng/mL to 100 µg/mL and assayed by MTT [3-(4,5-dimethylthiazolyl-2)-2, 5-diphenyltetrazoliumbromide], 72 hours after initial treatment. IC50 values were calculated with the PharmacoGx R package calculation [2]. The observed sensitivity and resistance to polatuzumab vedotin were in line with two previous studies [3,4].
Synergism assessment was done by exposing cells for 72 hours to increasing doses of polatuzumab vedotin and of disulfiram. The benefit of the combination was assessed both as synergism according to the Highest single agent (A, HSA, synergistic: HSA>10, additive: -10<HSA<10, antagonistic: HSA<-10) (A) and as potency (B, synergistic: potency>0.5, additive: 0<potency<0.5, antagonistic: potency<0) (B) and efficacy (C, synergistic: efficacy>1, additive: 0<efficacy<1, antagonistic: efficacy<0) according to the MuSyC algorithm [5].

CD79B expression

CD79B RNA expression values were extracted from the dataset GSE221770, previously produced via total-RNA-Seq [6].
Flow cytometry was performed on lymphoma cell lines to measure CD79b, CD19, and CD20 cell surface expression. Cells were washed with FACS buffer (PBS + 0.5% BSA) and divided into $1x10^6$ cells/tube.

A pretreatment with human FcR blocking (Miltenyi Biotec Inc., Auburn, CA, USA) was performed according to the manufacturer's instructions. CD19 PEcy7 (Beckman Coulter, 5µl;), CD79b APC (BD Biosciences, 5 µl), CD20 Pacific Blue (BioLegend, 5 µl) or related isotype controls were incubated with cells at RT for 15 minutes. Eventual dead cells were excluded by 7-AAD staining (BD Biosciences). Cells were washed twice in FACS buffer and re-suspended in FACS buffer. Flow-cytometry analysis was performed with a FACS Canto II instrument (BD Biosciences). Each sample's median fluorescence intensity (MFI) was determined using FacsDiva v8.0.1 software (BD Biosciences, Allschwil, Switzerland). Cells stained with isotype control antibody were used as controls.

References

1. Tarantelli C, Wald D, Munz N, Spriano F, Bruscaggin A, Cannas E, et al. Targeting CD19-positive lymphomas with the antibodydrug conjugate loncastuximab tesirine: preclinical evidence of activity as a single agent and in combination therapy. Haematologica. 2024;109(10):3314-3326.
2. Smirnov P, Safikhani Z, El-Hachem N, Wang D, She A, Olsen C, et al. PharmacoGx: an R package for analysis of large pharmacogenomic datasets. Bioinformatics. 2016;32(8):1244-1246.
3. Pfeifer M, Zheng B, Erdmann T, Koeppen H, McCord R, Grau M, et al. Anti-CD22 and anti-CD79B antibody drug conjugates are active in different molecular diffuse large B-cell lymphoma subtypes. Leukemia. 2015.
4. Kawasaki N, Nishito Y, Yoshimura Y, Yoshiura S. The molecular rationale for the combination of polatuzumab vedotin plus rituximab in diffuse large B-cell lymphoma. Br J Haematol. 2022;199(2):245-255.
5. Meyer CT, Wooten DJ, Paudel BB, Bauer J, Hardeman KN, Westover D, et al. Quantifying Drug Combination Synergy along Potency and Efficacy Axes. Cell Syst. 2019;8(2):97-108 e116.
6. Johnson Z, Tarantelli C, Civanelli E, Cascione L, Spriano F, Fraser A, et al. IOA-244 is a Non-ATP-competitive, Highly Selective, Tolerable PI3K Delta Inhibitor That Targets Solid Tumors and Breaks Immune Tolerance. Cancer Res Commun. 2023;3(4):576-591.
7. Ennishi D, Jiang A, Boyle M, Collinge B, Grande BM, Ben-Neriah S, et al. Double-hit gene expression signature defines a distinct subgroup of germinal center B-cell-like diffuse large B-cell lymphoma. Journal of Clinical Oncology. 2019;37(3):190-201.
8. Ianevski A, Giri AK, Aittokallio T. SynergyFinder 3.0: an interactive analysis and consensus interpretation of multi-drug synergies across multiple samples. Nucleic Acids Res. 2022;50(W1):W739-W743.
9. Zheng S, Wang W, Aldahdooh J, Malyutina A, Shadbahr T, Tanoli Z, et al. SynergyFinder Plus: Toward Better Interpretation and Annotation of Drug Combination Screening Datasets. Genomics Proteomics Bioinformatics. 2022;20(3):587-596.

Supplementary Figures and Tables

Figure S1. Spearman correlation between the *in vitro* anti-proliferative activities of polatuzumab vedotin and the expression of its target CD79B measured via total RNA-Seq data. CD79B RNA expression values were extracted from the dataset GSE221770, previously produced via total-RNA-Seq [6]. Correlation analyses were performed using R version 4.4.1 (2024-06-14).

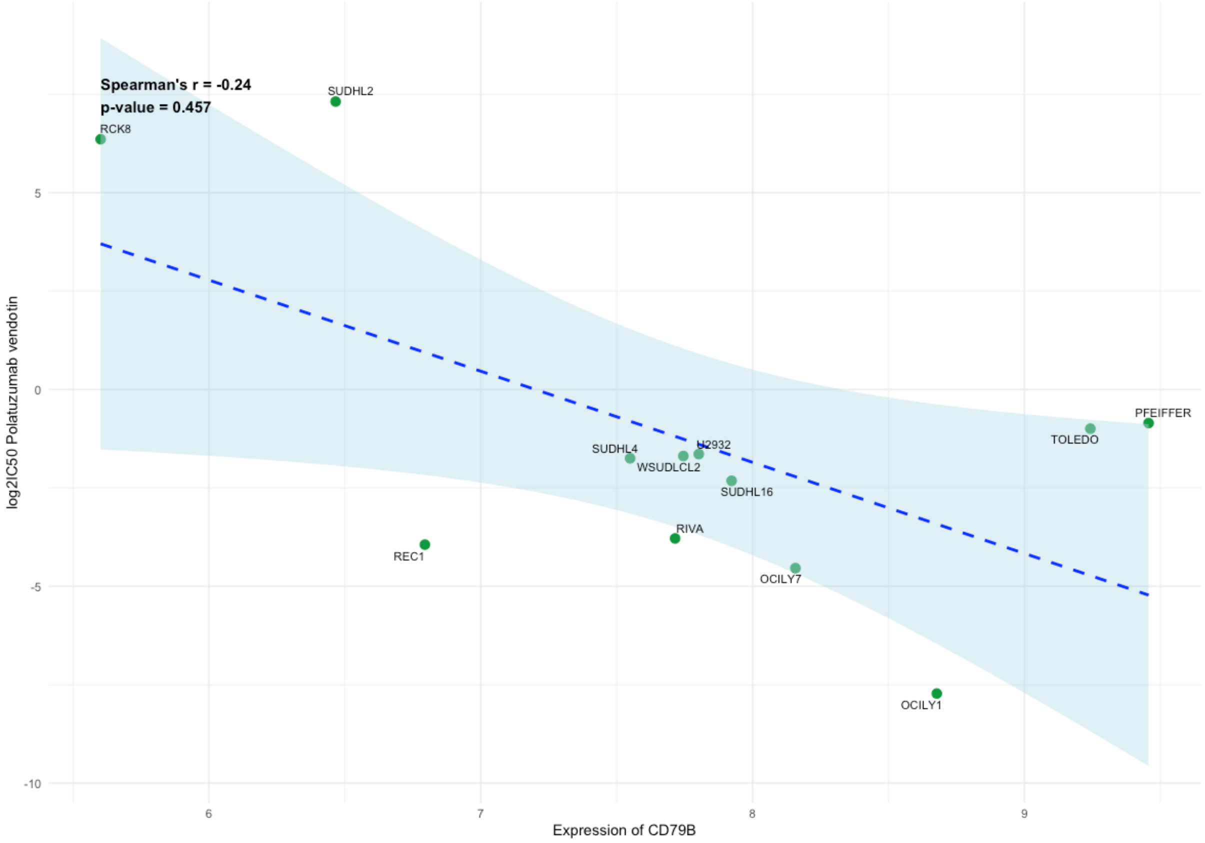

Figure S2. Pearson correlation between the *in vitro* expression of CD79B and ALDH1L1 measured via total RNA-Seq. RNA expression values were extracted from the dataset GSE221770, previously produced via total-RNA-Seq [6]. Correlation analyses were performed using R version 4.4.1 (2024-06-14).

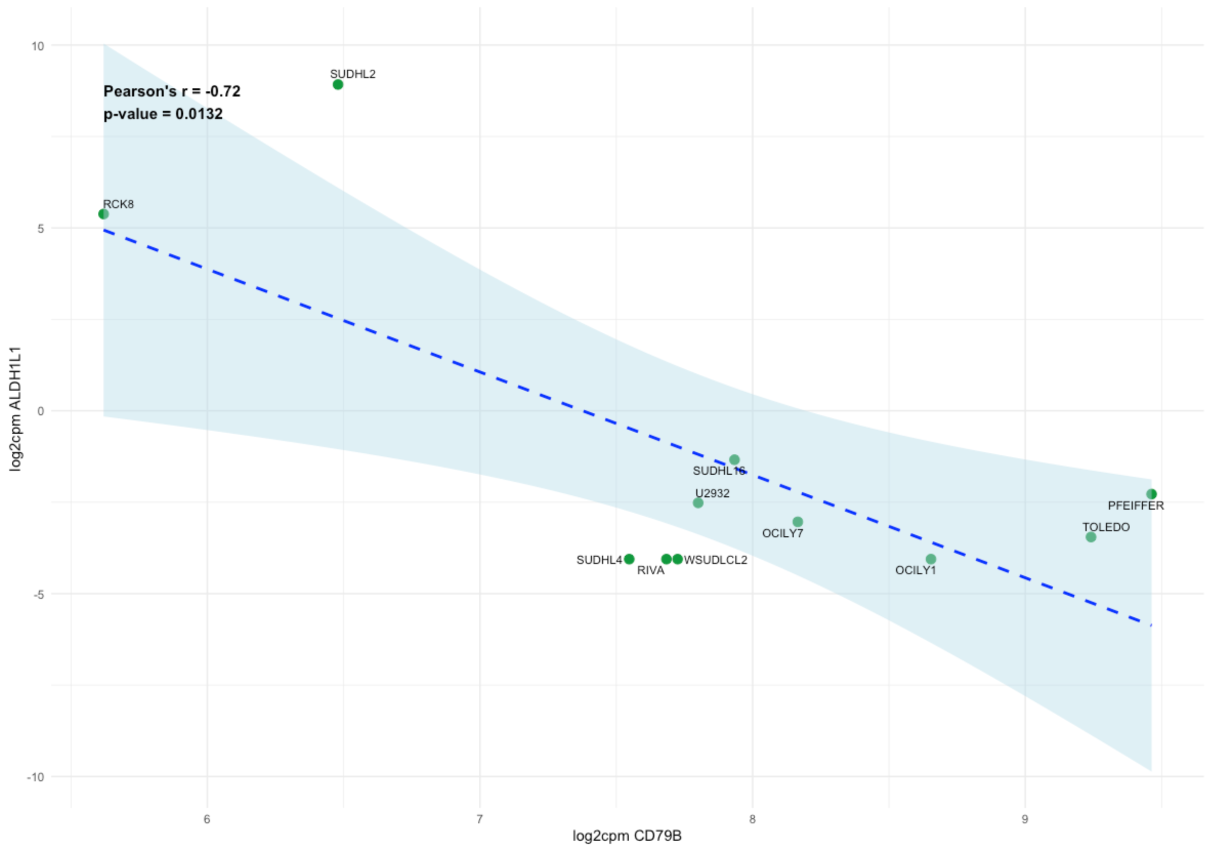

Figure S3. Pearson correlation between the *in vivo* expression of CD79B and ALDH1L1 measured via total RNA-Seq. RNA expression values were extracted from the Scott dataset [7]. Correlation analyses were performed using R version 4.4.1 (2024-06-14).

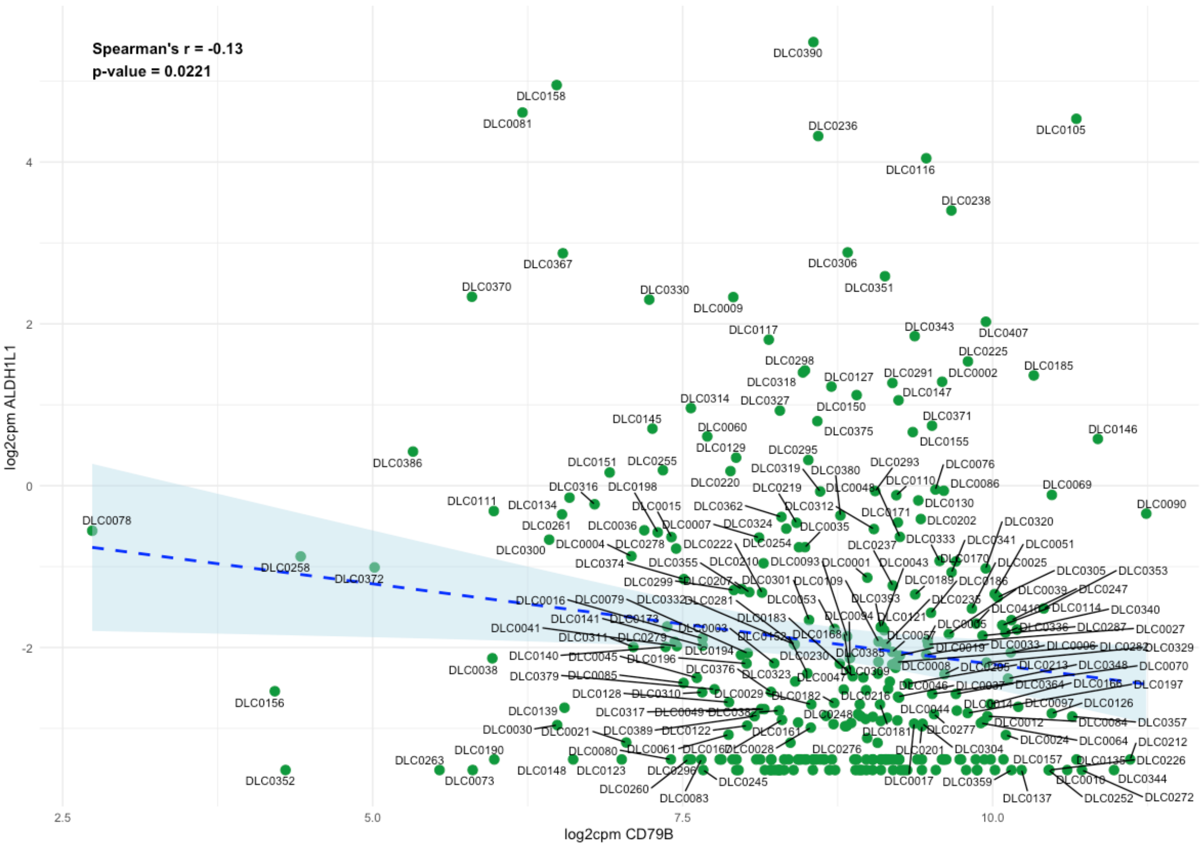

Figure S4. Combination of polatuzumab vedotin with the ALDH1 inhibitor disulfiram. Cell viability was assessed by MTT assay upon 72 hours of exposure. Boxplots show the median of at least three independent experiments. The benefit of the combination was assessed both as synergism according to the (A) Highest Single Agent (HSA, synergistic score>10, additive -10<score<10, antagonistic score< -10) model [8], calculated using the SynergyFinder R package [9], and as efficacy (B; synergistic: efficacy>1, additive: 0<efficacy<1, antagonistic: efficacy<0) according and potency (C; synergistic: potency>0.5, additive: 0<potency<0.5, antagonistic: potency<0) according to the MuSyC algorithm [5].

A

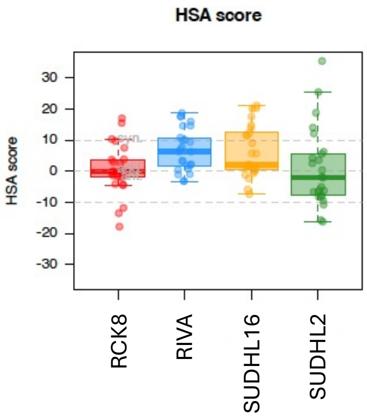

B

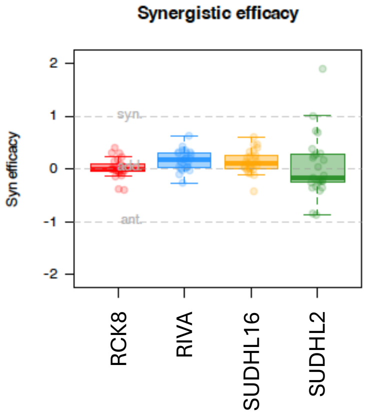

C

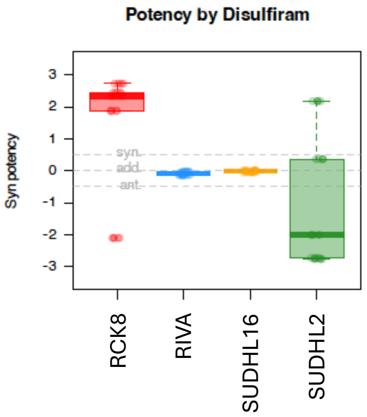

Supplementary Table 1. Supervised analysis of polatuzumab vedotin-resistant versus polatuzumab vedotin-sensitive cell lines. Limma analysis of the dataset GSE221770, previously produced via total-RNA-Seq [6]. Differentially expressed genes were identified by applying a moderated t-test (*limma* package in R environment).
Available at
https://www.dropbox.com/scl/fi/uzyk07lnpd8qha0rt93vy/TableS1_limma.xlsx?rlkey=5ewp0p7gcexgeilq3drpahlfe&e=1&st=9i9dmc9v&dl=0

Table S2. Flow cytometry results representing the ratio of median fluorescence of each marker to isotype control for SU-DHL-2 (S1A) and SU-DHL-16 (S1B). Cells were exposed for 48 hours to DMSO, 200 nM or 400 nM disulfiram before flow cytometry analysis.

S1A

**SU-DHL-2**	**CD19**	**CD79b**	**CD20**
DMSO	4	3	134
Disulfiram 200 nM	4	2	128
Disulfiram 400 nM	4	2	126

S1B

**SU-DHL-16**	**CD19**	**CD79b**	**CD20**
DMSO	0	51	1
Disulfiram 200 nM	0	54	1
Disulfiram 400 nM	0	51	1
